# H_2_S-Sensing Studies Using Interdigitated Electrode with Spin-Coated Carbon Aerogel-Polyaniline Composites

**DOI:** 10.3390/polym13091457

**Published:** 2021-04-30

**Authors:** Aamna Bibi, Yuola Rose M. Rubio, Karen S. Santiago, His-Wei Jia, Mahmoud M. M. Ahmed, Yi-Feng Lin, Jui-Ming Yeh

**Affiliations:** 1 Center for Nanotechnology, Department of Chemistry, Chung Yuan Christian University (CYCU), Chung Li District, Tao-Yuan City 32023, Taiwan; emi2118@gmail.com (A.B.); yuolarosemorenorubio@gmail.com (Y.R.M.R.); itri920314@yahoo.com.tw (H.-W.J.); mahmoud.ahmed@mail.ntust.edu.tw (M.M.M.A.); 2 Department of Chemistry, College of Science, University of Santo Tomas, Espana, Manila 1015, Philippines; kssantiago@ust.edu.ph; 3 Department of Chemical Engineering and Center for Nanotechnology at CYCU, Chung Li District, Tao-Yuan City 32023, Taiwan

**Keywords:** polyaniline, carbon aerogel, composite, H_2_S gas sensor, room temperature

## Abstract

In this paper, carbon aerogel (CA)-polyaniline (PANI) composites were prepared and first applied in the study of H_2_S gas sensing. Here, 1 and 3 wt% of as-obtained CA powder were blended with PANI to produce composites, which are denoted by PANI-CA-1 and PANI-CA-3, respectively. For the H_2_S gas-sensing studies, the interdigitated electrode (IDE) was spin-coated by performing PANI and PANI-CA composite dispersion. The H_2_S gas-sensing properties were studied in terms of the sensor’s sensitivity, selectivity and repeatability. IDE coated with PANI-CA composites, as compared with pristine PANI, achieved higher sensor sensitivity, higher selectivity and good repeatability. Moreover, composites that contain higher loading of CA (e.g., 3 wt%) perform better than composites with lower loading of CA. At 1 ppm, PANI-CA-3 displayed increased sensitivity of 452% at relative humidity of 60% with a fast average response time of 1 s compared to PANI.

## 1. Introduction

Hazardous gases may exist in an indoor or outdoor environment. Therefore, analysis and monitoring of toxic gases such as H_2_S, SO_2_ and NO_x_ are required. Hydrogen sulphide gas (H_2_S) is a colourless, flammable, poisonous and corrosive gas. It is usually produced from the bacterial breakdown of organic matter by the absence of oxygen gas and the decomposing wastes of humans and animals. Exposure to this gas can lead to critical health issues in humans. Thus, smart devices such as gas sensors are still an important area of development to avoid risks [1,2,3]. A growing body of research is focusing on the fabrication of robust, portable and low-cost gas sensors. These sensors play an important role in the medical field, environmental monitoring, industrial safety control and security [4,5].

Among the array of conducting polymers (CP), polyaniline (PANI) is considered highly stable, with a unique redox chemistry and the ability to electrically switch between its conductive and resistive states through the doping/dedoping process, which can be controlled by acid/base reaction [6,7,8]. The redox property of PANI and its derivative polymers make them applicable for electrochemical sensing [9,10,11]. Their doping/dedoping property also expands their useability for gas sensing [12,13,14]. The first publication related to H_2_S sensing was reported by Monkman in 1995.

However, PANI suffers from low electrical conductivity due to insufficient conducting pathways in its matrix and inconsistency in properties [15]. Hence, significant attention has focused on coupling PANI with other heterogeneous species, which has resulted in many remarkable studies [16]. Among the spectrum of heterogeneous fillers, carbon-based materials are typically used to extend the functionality, overcome the poor processability and improve the conductivity of PANI. These carbon-based fillers may enhance the sensitivity and selectivity of PANI-based sensor via electronic interaction, charge transfers or morphological modification.

In the past decades, carbon aerogels (CA) attracted intensive and extensive research interest. CA are highly cross-linked nanosized porous materials. Being light weight, mesoporous and conductive with a large surface area, CA are being employed in different fields, including supercapacitors, advanced catalyst supports, absorbents, rechargeable batteries, environmental protection and chromatographic packing [17,18,19,20,21,22,23,24,25,26]. CA are formed by pyrolysis of organic aerogels at temperatures of 800–900 °C [27]. During this procedure, organic aerogels transform into a carbon network with good electrical conductivity as high as 0.1–1 S/cm [28,29].

To our knowledge, no report has dealt with CA-filled polymer-based conductive composites as a candidate for application in H2S sensing. Therefore, in this study, we report the preparation of PANI-CA composites and first applied it in H2S sensing on the interdigitated electrode (IDE) of ITO glass. The PANI-CA composites were prepared by incorporating different loadings of CA dispersed in NMP and spin-coated onto the IDE as a gas sensor. The physical and chemical properties of the composites were analysed, and the gas-sensing characteristics were studied in terms of sensitivity, selectivity and repeatability.

## 2. Materials and Methods

Aniline (99%, Alfa aesar, Lancashire, UK) distilled before use, ammonium peroxodisulfate (APS) (J. T. Baker, Randor, PA, USA)), N-methyl-2-pyrrolidone (uniregion bio-tech, Taoyuan, Taiwan), Resorcinol (98%, Sigma Aldrich, Saint Louis, MI, USA), hydrochloric acid (HCl, 37%, Honey well Riedel-de Haen, New Taipei, Taiwan), sodium carbonate (99.5%, Sigma Aldrich, Saint Louis, MI, USA), ammonium hydroxide solution (NH_4_OH, 28%, Honey well Riedel-de Haen, New Taipei, Taiwan) and ethanol (ECHO, Miaoli, Taiwan) were used as received without further purification.

DMSO-d6 was used to perform ^1^H-NMR spectra on a Bruker 300 spectrometer. FTIR spectra ranging from 4000 to 650 cm^−^^1^ were recorded on FT/IR-4100 spectrometer at a resolution 4.0 cm^−^^1^. Scanning electron microscopy (SEM), (Hitachi S-2300) was used for examining the surface morphologies of the materials while the surface area and pore volume were determined by N_2_ adsorption–desorption isotherm (BET). Hitachi U-2000 UV-Visible spectrometer and Waters GPC-150CV (Waters, Shanghai, China) were used for UV-Vis spectroscopy and molecular weight determination. Electro-spinning facility and gas-sensing devices were constructed in our lab.

### 2.1. Synthesis of CA

**Scheme 1:**
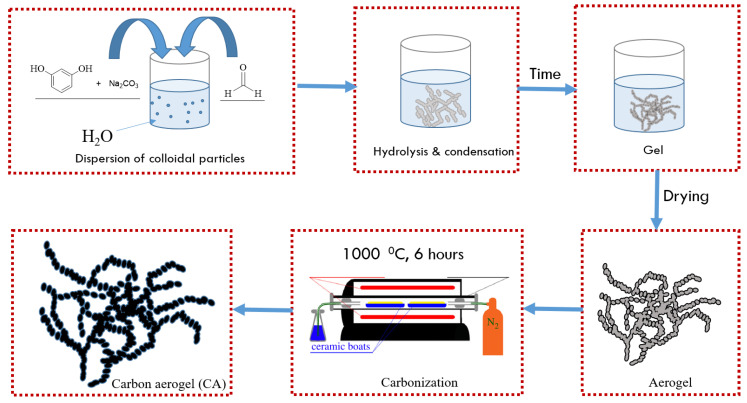
Schematic diagram for the preparation of PANI-CA composite.

Scheme 1 shows the schematic for the preparation of CA. First, resorcinol (1 M) and sodium carbonate (1.32 × 10^−3^) were mixed in water and stirred for 5 min at room temperature. Then, formaldehyde (1.32 M) was added and stirred for 1 h. Afterwards, the resulting solution was kept for two days (first day at 45 °C, second day at 75 °C) for hydrolysis and condensation reactions. At this point, the process of cross-linking had finished and gel was formed. The obtained gel was subsequently immersed in ethanol for five days, with the ethanol being replaced each day to remove the residual solvents in the gel. The gel was stored at 25 °C for 7 days to remove excess ethanol. Lastly, the carbon aerogel was obtained by carbonisation at 1000 °C for 6 h under a nitrogen environment [30].

### 2.2. Preparation of Carbon Aerogel-Based Polyaniline Composites

The carbon aerogel-based PANI composites were prepared by a simple physical mixing method, as a representative procedure. First, 1 wt% of the emeraldine base form of PANI fine powder was dissolved in NMP and followed by magnetic stirring for 3 h. Subsequently, 1 and 3 wt% of CA fine powder (with respect to PANI) were introduced into the previous PANI solution under stirring, followed by sonicating for 2 h. The as-obtained composites were denoted by PANI-CA-1 and PANI-CA-3, respectively.

### 2.3. Preparation of PANI and PANI/CA Sensor

**Scheme 2:**
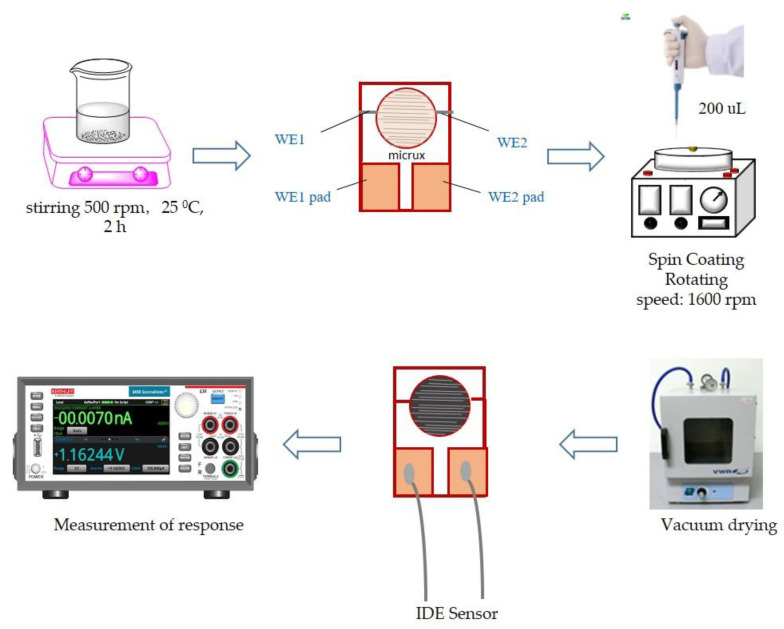
Schematic steps of the gas sensor fabrication process.

In this study, the IDE coated with PANI and PANI-AC composite dispersion was constructed by spin-coating technique, as shown in Scheme 2. PANI, PANI-AC-1 and PANI-AC-3 solutions (1 wt%) were prepared by dissolving 0.1 gm of the respective sample in 10 gm of NMP by stirring at room temperature. Afterwards, thin films were prepared by spin-coating a 200 µL solution on an IDE at a rotation speed of 1600 rpm. The IDE sensor was then dried in a vacuum oven prior to use in gas sensing.

### 2.4. Gas Sensing

**Scheme 3:**
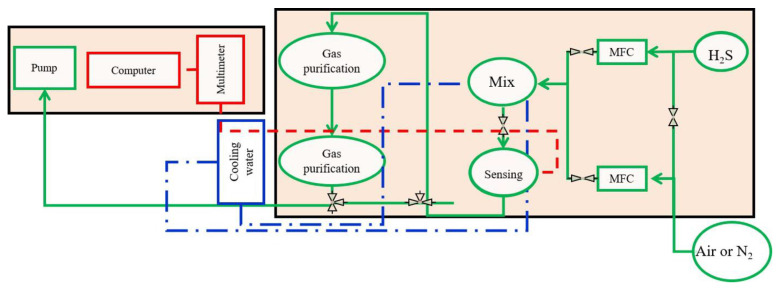
Illustration of the setup for H_2_S sensing measurement.

The H_2_S gas-sensing property was studied via a gas sensor setup, which is shown in Scheme 3. The experiment was initiated by exposing the sensor to N_2_ gas [31,32] to attain the steady state, followed by exposure to H_2_S gas concentration ranging from 1 to 50 ppm for 2 min. All the experiments were performed at room temperature (25 ± 0.5 °C) at two relative humidity (RH%) of 60% RH and 80% RH by applying a fixed voltage of 0.1 V (1000 sccm). The change in electrical conductivity [33,34,35,36] of a PANI and PANI-CA composite was used to the determine the gas response as follows: [37,38]

(1) $$Sensor^{\prime }sresponse=\frac{I}{I_{0}}$$

where *I*_o_ and *I* represent the conductance of the material in N_2_ gas and upon exposure to H_2_S gas, respectively.

## 3. Results

The base form of PANI synthesised by oxidative chemical polymerisation can be schematically represented by the following general formula:
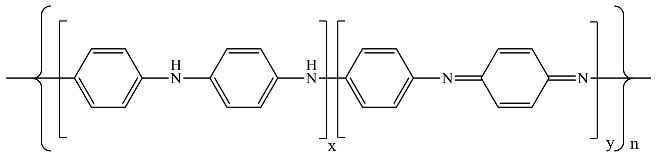
where x + y = 1, when x = 1, y = 0 for the fully reduced polymer (so-called leuco-emeraldine); x = 0, y = 1 for the fully oxidised polymer (so-called pernigraniline); and x = 0.5, y = 0.5 for the half-oxidised polymer (emeraldine). CA was fabricated by high-temperature carbonisation of as-prepared phenolic resin. A specific feeding ratio of CA was incorporated into the PANI matrix to prepare the CA-based PANI composites.

### 3.1. Characterisation

#### 3.1.1. Polyaniline (PANI)

Figure S1a shows the representative ^1^H-NMR spectrum (in DMSO-d_6_) of the conventional PANI. The chemical shift at 2.5 and 3.34 ppm was assigned to the solvent and residual water protons, respectively. The major signal centred at around 6.5–7.5 ppm was due to protons on phenylene and disubstituted phenylene units. The weak peak at 5.7 and 6.5 ppm was due to the (–NH– and –NH_2_) end group, respectively [39]. Figure S1b exhibits the representative FTIR spectrum. The characteristic peaks of PANI observed at 1584 and 1490 cm^−1^ correspond to quinoid ring stretching (-N=Q=N-) and benzene ring stretching (-N-B-N-), respectively, where Q represents a quinoid ring and B denotes a benzene ring. The adsorption peaks that appeared at 3255 and 1309 cm^−1^ correspond to the amine N-H stretching and C-N stretching vibration in the benzenoid ring, while the peak at 1163 cm^−1^ corresponds to the vibrational mode of protonated amines formed during acid doping. The peaks found at the position of 3155, 3021 and 823 cm^−1^ are attributed to C-H stretching vibration and out-of-plane bending vibration of the benzenoid ring [40]. The molecular weight of the as-prepared PANI was obtained by GPC analysis with the value of M_w_ = 76,700, M_n_ = 10,100 and PDI = 7.6, respectively.

#### 3.1.2. Carbon Aerogel (CA)

**Figure 1:**
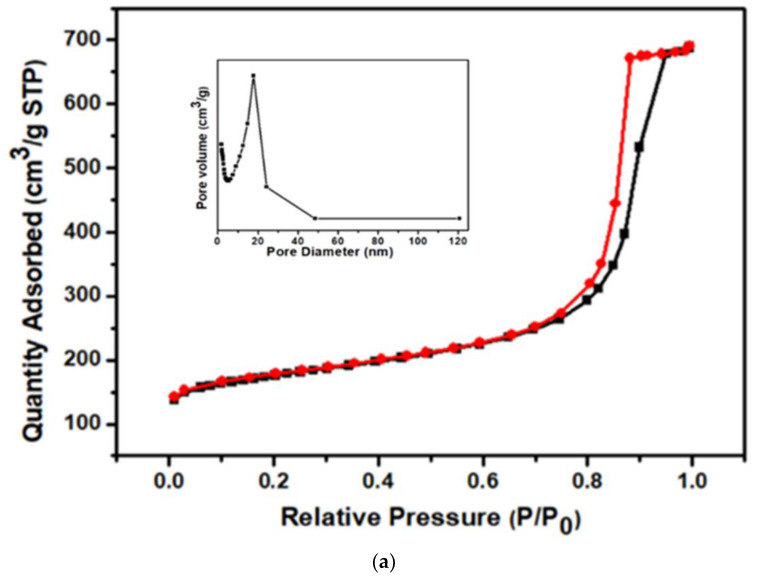

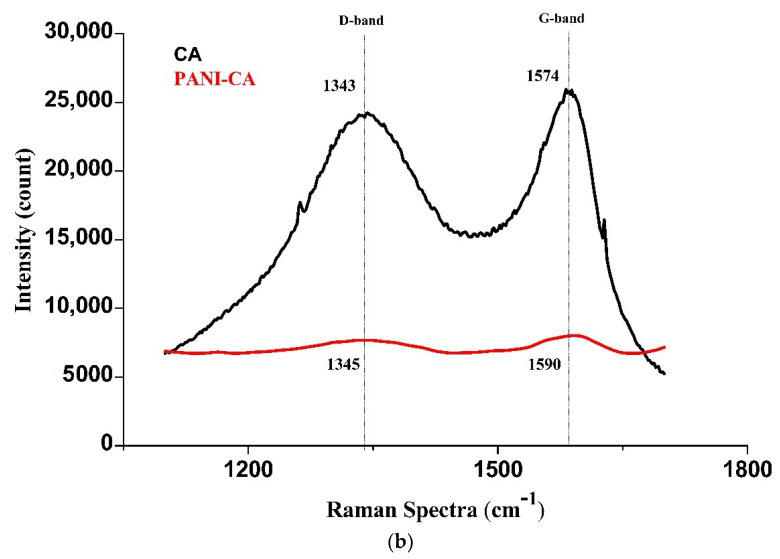
The representative figure of (**a**) BET curve and (**b**) Raman spectrum of CA.

**Figure 2:**
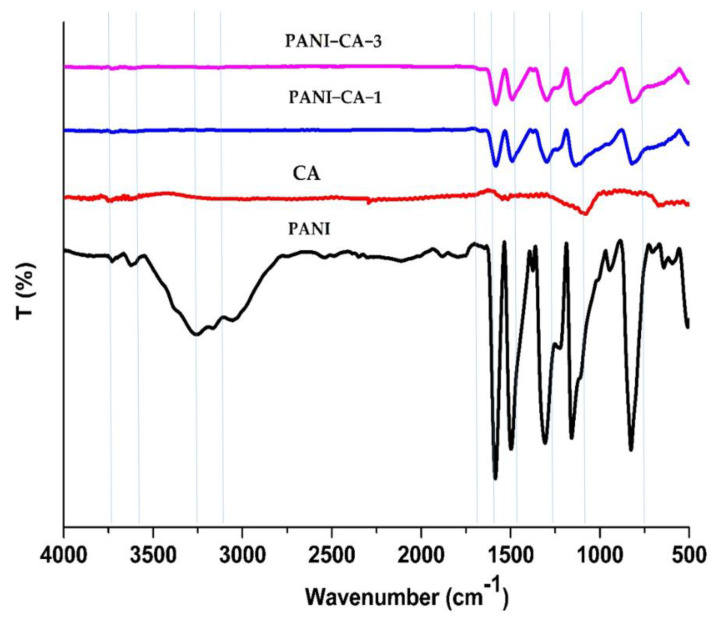
The representative FTIR spectra of PANI, CA, PANI-CA-1 and PANI-CA-3.

CA was successfully synthesised through atmospheric drying followed by carbonisation. The N_2_ adsorption–desorption isotherm of CA is shown in Figure 1a. The isotherm was type IV, indicating the dominant effect of capillary condensation phenomenon on the surface of CA. A smaller and sharper hysteresis loop indicates a narrower pore size distribution. When the relative pressure was greater than 0.8, the adsorption quantity increased sharply. The hysteresis loops indicated the coexistence of microspores and mesoporous in the structure. The inset in Figure 2 shows the pore size distribution of the CA. The pore size distribution focused on 19 nm and the reduced pore size resulted in a more compact network structure [41,42], which was consistent with the SEM images. The specific surface area of as-prepared CA was ~724 m^2^/g [30].

Raman spectroscopy was performed to identify the extent of the disorder of CA. As shown in Figure 1b, two peaks were observed in neat CA. The peak appeared at the 1343 cm^−1^ (D-band) and 1574 cm^−1^ (G-band) for C-C bonds, corresponding to the defects or partially disordered structure of the carbon domain and conjugated bond, respectively. For the PANI-CA composite, the same peaks were observed with a slight shift in G-band, implying the formation of a new covalent bond. Moreover, the intensity ratio between D and G band (I_D_/I_G_) was 0.849 and 0.845 for CA and PANI-CA composites, respectively, indicating a good similarity between the two products [43,44].

#### 3.1.3. Composites

The Fourier-transform infrared spectroscopy (FTIR) spectra of PANI, CA, PANI-CA-1 and PANI-CA-3 are shown in Figure 2. The peak at 3741 cm^−1^ can be attributed mainly to the –OH groups bonded to the benzene ring but also may be due to –CH_2_OH groups connected to the resorcinol molecule, which did not take part in network formation. The small peak appears at 3361 cm^−1^ can be correlated to the primary OH groups. The peak at 1588 cm^−1^ is due to aromatic ring stretching. The peaks observed at 1091 cm^−1^ confirm the C-O-C linkage stretching between the two resorcinol molecules. Because of heating, some of the CH_2_-O-CH_2_ linkages may have broken to form CH_2_OH and =CH_2_ groups attached to different resorcinol molecules. This is further supported by the peak observed at 668 cm^−1^ due to the C=CH_2_ groups.

All the characteristic peaks of PANI and CA appeared in the FTIR spectrum of PANI-CA-1 and PANI-CA-3, which is attributed to the covering of PANI network on the surface of CA. However, due to the incorporation of CA in the plane matrix, a slight shift in the characteristic bands occurred. The slight shift in the observed bands indicated the presence of interaction between PANI and CA. Viewed from the blue line region of FTIR spectra, there exist peaks (near 3272 cm^−1^, caused by –NH– group) in the curves of PANI and PANI-CA, but not CA. Furthermore, for the CA-based PANI composites, all the typical peaks of PANI were apparent with less intensity, thereby clearly revealing the presence of CA.

### 3.2. Morphological Observations of PANI, CA and Composite

**Figure 3:**
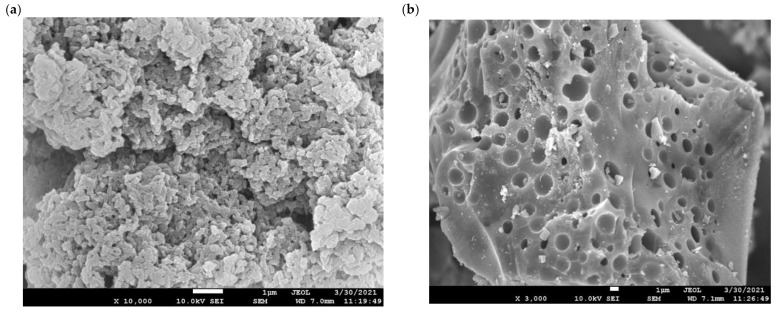

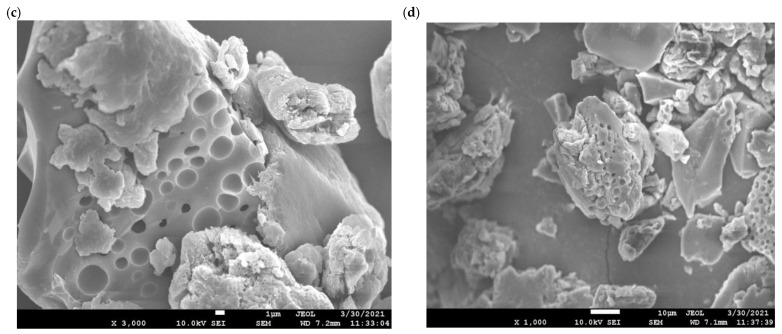
The representative morphological images of SEM for (**a**) PANI (**b**) CA (**c**) PANI-CA-1 and (**d**) PANI-CA-3.

In investigating the surface morphologies, the SEM observations of PANI, CA and their composites were studied, as shown in Figure 3.

The SEM image of PANI (Figure 3a) film generates a characteristic granular morphology where the chain organisation is reduced and leads to the structure of conducting island. Ordered polymer chains are separated by disordered regions of low conductivity [45]. The image in Figure 3b shows the surface morphology of as-prepared CA, which confirmed that the polymeric gel structure resulted in the formation of mesoporous CA with a larger surface area. The network in CA clearly has pores, which have a diameter of 50 nm [46].

The morphology of PANI changed after CA was added, as shown in Figure 3c,d. No obvious large agglomeration was observed on the surface of PANI-CA, thereby indicating that PANI diffused into the mesopores of CA during the blending process. Figure 3c shows the SEM of PANI-CA-1 with fewer pores and CA was diffused into the PANI. PANI-CA-3 (Figure 3d) had a more porous blended surface, thereby leading to a much higher sensor response.

### 3.3. H_2_S Gas-Sensing Performance

#### 3.3.1. Gas-Sensing Mechanism

PANI is a p-type semiconductor in which the majority of charge carriers are holes. When it is exposed to a reducing gas such as H_2_S, a decrease in conductivity may be expected. However, an increase in conductivity was observed instead, which may have been caused by the presence of water vapours in the test chamber and sensing layer. H_2_S can react with water molecules and ionise into H^+^ and HS^-^, where H^+^ ion may have doped the PANI [12,47], thus increasing the conductivity [13].

(2) $$H_{2}S+\mathrm{PANI}\;\to {\mathrm{HS}}^{-1}+{\mathrm{PANIH}}^{+}$$

During the recovery process, PANI had a longer recovery time due to the presence of water, preventing the H_2_S from escaping easily from the surface.

#### 3.3.2. Sensor’s Response and Sensitivity

**Figure 4:**
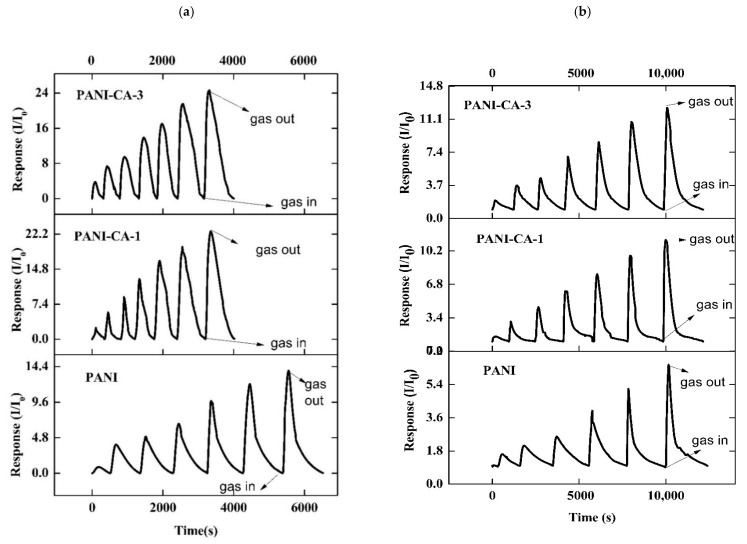
Time-dependent response of PANI, PANI-CA-1 and PANI-CA-3 to H_2_S gas ranging from 1 to 50 ppm (1000 sccm) at two RH% (**a**) 60% and (**b**) 80%.

Figure 4 shows the transient response of PANI and its composites at two RH % to H_2_S as a function of time to highlight the effect of filler on the gas-sensing properties. IDE coated with PANI, PANI-CA-1 and PANI-CA-3 switched between H_2_S (1–50 ppm) and N_2_ as exposure and recovery duration. The response was determined in terms of conductivity by applying a fixed voltage of 0.1 V. Generally, the response of all sensors increased with the increase in gas concentration, with the smallest response at 1 ppm and the highest response at 50 ppm. The H_2_S-sensing properties of all the sensors were compared at room temperature. A marked difference can be seen in the response behaviour of a sensor with and without the filler, i.e., CA.

At 60% RH the response values (Figure 4a) for PANI without any filler were 0.84, 3.88, 4.95, 6.69, 9.77, 12.04 and 13.83 at corresponding gas concentrations of 1, 5, 10, 20, 30, 40 and 50 ppm, respectively. For PANI-CA-1, the observed response was in the order of 2.35, 5.64, 8.89, 12.71, 16.57, 19.51 and 22.75 towards H_2_S concentration ranging from 1–50 ppm. The response of PANI-CA-1 sensor was two times higher than that of PANI on average. In addition, a much higher response can be seen in the case of PANI-CA-3 with response values of 3.80, 7.37, 9.50, 13.87, 17.01, 21.57 and 24.64, which on average are 2.24 and 1.2 times higher than those of PANI and PANI-CA-1, respectively. This marked increase in sensitivity response may be associated with the large surface area of CA. The relative response increased due to the availability of a large number of active sites in the sensing layer. In our experiment, the lower detection limit for the sensors was 1 ppm. At a higher concentration rate of increase, the sensor’s response slows down probably due to the less availability of active sites on the surface due to the adsorption of gas molecules. Hence, the performance of sensors increased in the order PANI < PANI-CA-1 < PANI-CA-3.

It has been observed that the sensor’s response is strongly dependent on the ambient RH. The PANI sensor showed response values of 1, 1.6, 2, 3, 4, 5 and 6.5 in 80% RH (Figure 4b) towards H_2_S at corresponding concentrations of 1, 5, 10, 20, 30, 40 and 50 ppm respectively. While the PANI-CA-1 sensor showed a response value of 1.5, 3, 4, 6, 8, 9.7 and 11.3 in 80% RH at the same concentration, which was 1.85 times higher than PANI on average. Similarly PANI-CA-3 sensor showed response values of 2, 3.7, 4.5, 6.9, 8.5, 10.7 and 12.3 in 80% RH towards H_2_S gas concentration ranging from 1–50 ppm respectively. The response observed in case of PANI-CA-3 sensor was 1.2 times and 2.3 times higher than PANI-CA-1 and PANI respectively.

However, we found that the sensor conductivity and response to H_2_S are dramatically decreased with increasing humidity. In a humid environment, different types of interactions can occur when water vapour is adsorbed on the sensor’s surface such as swelling, physical entrapment due to high porosity, electron withdrawing and hydrogen bonding etc. In our experiments, it was observed that after increasing the humidity inside the chamber, the conductivity of the sensors was decreased. Since the water molecules are polar and can act as a weak acid, they can dope PANI, and as a result increase the conductivity. Therefore, as the RH increases, the sensitivity of the as prepared sensors to H_2_S decreases. When RH is very high such as 80%, the PANI molecules are highly doped and H_2_S molecules interact with H_2_O molecules instead of PANI since no more room exists for further doping in the PANI [47].

**Figure 5:**
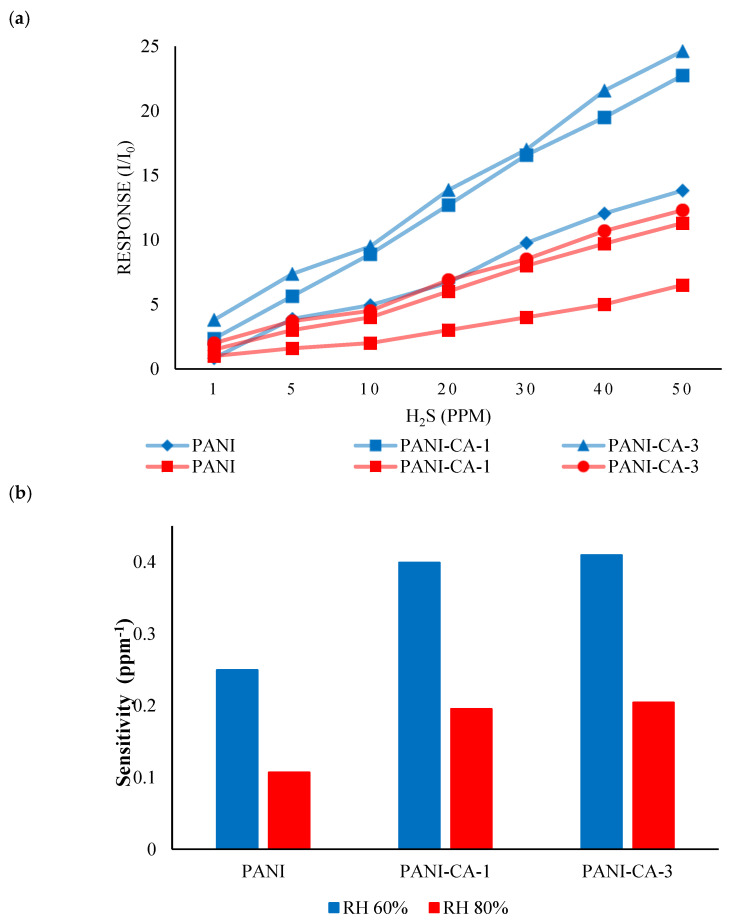
(**a**) Response curve of PANI, PANI-CA-1, PANI-CA-3 sensor (60% RH (blue) and 80% RH (red)) to increasing concentration of H_2_S (V = 0.1 V). (**b**) The sensor’s sensitivity values were reported in the bar plot.

The linear calibration curve in Figure 5a shows the response of these gas sensors as a function of H_2_S concentration. The linear fitting equations (at 60% RH) are determined as y = 0.2293x + 1.8732, y = 0.3992x +3.7348 and 0.4096x + 4.8369 for the PANI, PANI-CA-1 and PANI-CA-3 sensors, respectively. The correlation coefficients of the fitted data (R^2^) are 0.9766, 0.9786 and 0.9887, respectively (Table 1). While at 80% RH, the observed linear fitting equations for PANI, PANI-CA-1 and PANI-CA-3 are y = 0.107x + 0.9164, y = 0.1952x + 1.8639 and y = 0.2042x + 2.393 respectively. Moreover, the sensor’s sensitivity (S, [ppm^−1^]) was calculated as the slope of the normalised sensor response I/I_0_ as shown in Figure 5b. Evidently, the PANI-CA-1 and PANI-CA-3 sensors exhibited better sensitivity in comparison with the PANI sensor. For PANI, at 60% RH the sensitivity was 0.2493, which increased to 0.3992 (1.6 times) for PANI-CA-1. The highest sensitivity was found in the PANI-CA-3 sensor, with a value of 0.4096 (Table 1). Also, at 80% RH the highest sensitivity (0.2042 ppm^−1^) value was observed for PANI-CA-3, which was 1.05 times and 1.91 times higher than PANI-CA-1 and PANI respectively. Hence, the optimal amount of CA filler in PANI-CA composite was 3 wt%.

#### 3.3.3. Response/Recovery Time

**Figure 6:**
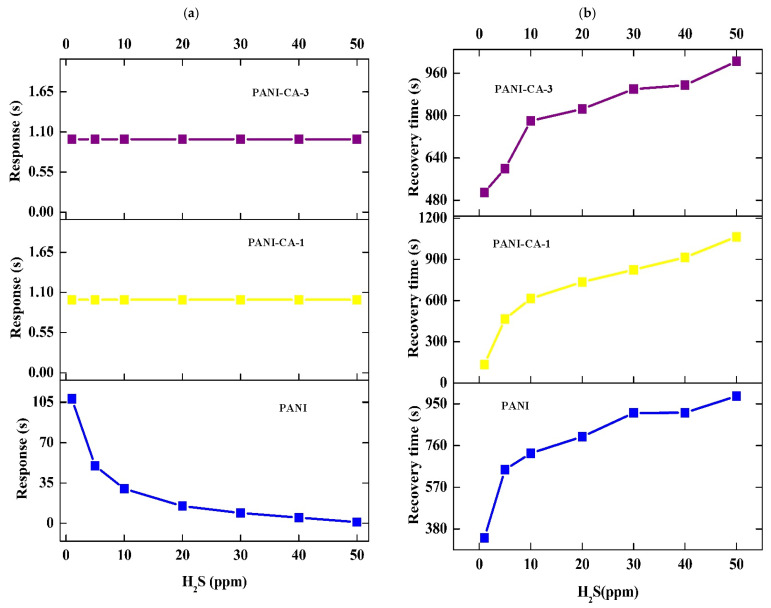
Dynamic quick response time (**a**) and recovery time (**b**) curves of PANI, PANI-CA-1 and PANI-CA-3 sensors when exposed to various concentrations of H_2_S at room temperature (60% RH).

Another factor that characterises a gas sensor is response/recovery time. When the sensor is exposed to gas, adsorption and desorption take place simultaneously. Therefore, response and recovery time depends solely on the relative adsorption/desorption rate. Figure 6 shows the dynamic response curves for the response/recovery time of all IDE sensors as a function of H_2_S gas concentration (60% RH). Figure 6a shows that the quick response time for PANI was in the range of 1 s to 108 s (±0.05) as the gas concentration increased from 1 to 50 ppm. On the other hand, the response time reduced to just 1 s for PANI-CA-1 and PANI-CA-3 at all concentrations with the addition of CA. The fast response time for composites may be attributed to the higher absorption rate due to a larger surface area as compared with PANI. On the other hand, the recovery time for hybrid composites was in the range of 135 s to 1065 s (±0.05), while that for the PANI was 340 s to 985 s, as shown in Figure 6b. At 50 ppm, IDE coated with PANI-CA-3 and PANI-CA-1 showed a longer recovery time of 1065 and 1005 s (±0.05), respectively, as compared with PANI, with a recovery time of 985 s. The poor recovery time for composites may be attributed to the high surface area, which allows the absorption of a sufficient amount of H_2_S gas molecules to dope. Thus, dedoping the sensing material takes a longer time, i.e., the desorption rate is much slower than the absorption rate. The response/recovery data at 80% RH are presented in Table S2.

#### 3.3.4. Repeatability

**Figure 7:**
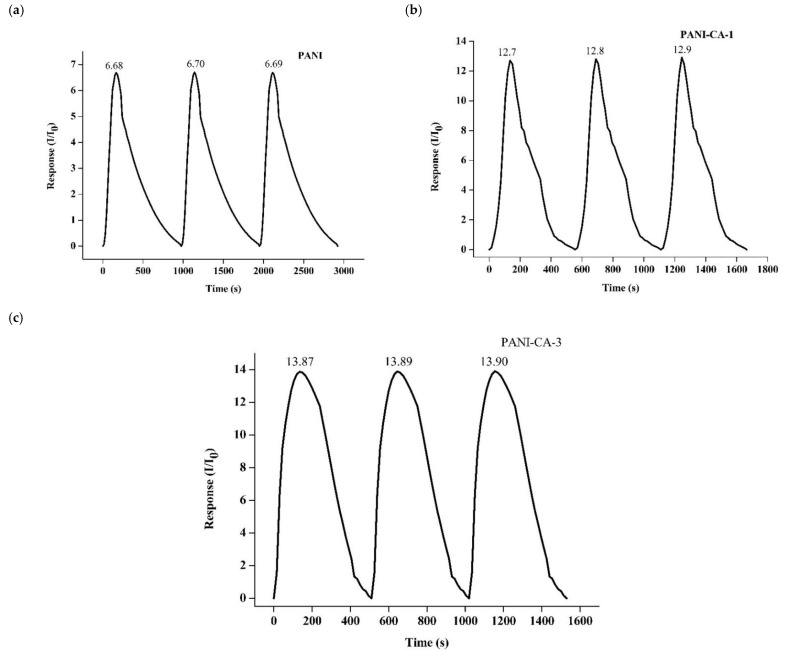
Repeatability test of PANI (**a**) PANI-CA-1 (**b**) PANI-CA-3 (**c**) sensor as normalised current (*I*/*I*_0_) to the same concentration of H_2_S (20 ppm) flowed through the measuring chamber (1000 sccm, V = +0.1 V) at 60% RH.

Repeatability is an important performance indicator of a gas sensor. For this part, all the IDEs coated with PANI and its composites underwent successive exposure to H_2_S at 20 ppm. Each test was performed three times at a 120 s interval in H_2_S gas at room temperature (Table S3). The sensors showed excellent repeatability with almost identical curves, as shown in Figure 7. All the sensors displayed a stable response with values of 6.69 ± 0.008, 12.8 ± 0.1 and 13.88 ± 0.01 for PANI, PANI-CA-1 and PANI-CA-3, respectively. However, the PANI-CA-3 sensor showed a stable response with the highest response value.

#### 3.3.5. Selectivity

**Figure 8:**
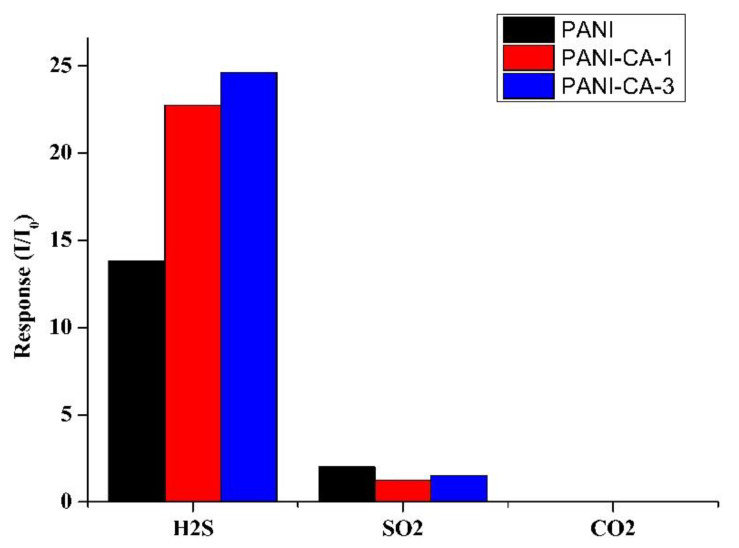
Selectivity study of PANI, PANI-CA-1 and PANI-CA-3 gas sensor towards H_2_S, SO_2_, CO_2_ at 50 ppm (1000 sccm) at 60% RH.

Selectivity is another salient feature for executing gas-sensing studies. It is the ability of a sensor to respond to a particular gas in the presence of other test gases and is an important parameter to determine the reliability of a gas sensor. In this part, IDE sensors coated with PANI, PANI-CA-1 and PANI-CA-3 were exposed to various gases, including H_2_S, SO_2_ and CO_2_, under the same concentration of 50 ppm at room temperature. Figure 8 shows the response curves of all sensors towards different gases. In general, all the sensors show a similar trend and exhibited a maximum response towards H_2_S as compared with other gases. In the case of H_2_S, PANI-CA-3 showed the highest response value of 2464% followed by PANI-CA-1, which had a response value of 2275%. The smallest response value of 1383% was observed for PANI. Hence, the selectivity of PANI and its composite-based sensors exhibited the following trend: PANI < PANI-CA-1 < PANI-CA-3. In contrast, all the IDE sensors showed low or almost no response towards SO_2_ and CO_2_.

## 4. Conclusions

We successfully prepared a PANI-CA composite by using the physical mixing method. FTIR, Raman spectroscopy and SEM revealed the presence of CA in the PANI matrix. The SEM images confirmed that the CA was uniformly dispersed on the surface of PANI. The prepared PANI-CA composite possesses a porous structure and surface defects, which help improve the gas-sensing properties of the prepared samples. From the gas-sensing results, the PANI-CA-based gas sensor can be concluded to have good sensing performance towards H_2_S gas at room temperature at concentrations ranging from 1–50 ppm. Moreover, the composites exhibit quick response and good reproducibility, indicating their promising application as gas-sensing materials. The response time was just 1 s upon exposure to H_2_S gas (60% RH). The most sensitive composite thin film to H_2_S gas was obtained by incorporating 3 wt% CA into the PANI matrix. Thus, PANI-CA porous composite-based gas sensor can be a good candidate for room temperature H_2_S sensing.

## Figures and Tables

**Table 1:** Sensor’s sensitivity of PANI at distinctive loadings of CA (0, 1, 3 wt%).

Sensor	RH %	Sensitivity (ppm^−1^)	SD	R^2^
PANI	60%	0.2293	0.007	0.9766
PANI	80%	0.107	0.008	0.9943
PANI-CA-1	60%	0.3992	0.01	0.9786
PANI-CA-1	80%	0.1952	0.001	0.9922
PANI-CA-3	60%	0.4096	0.008	0.9887
PANI-CA-3	80%	0.2042	0.007	0.9915

## Data Availability

No new data were created or analyzed in this study. Data sharing is not applicable to this article.

## Supplementary Materials

The following are available online at https://www.mdpi.com/article/10.3390/polym13091457/s1, Table S1. Quick response(s) and recovery time data of PANI (a) PANI-CA-1 (b) PANI-CA-3 (c) sensor as normalized current (I/I0) to the concentration ranging from 1 to 50 ppm flowed through the measuring chamber (1000 sccm, V = +0.1 V) at 60% RH, Table S2. Quick response(s) and recovery time data of PANI (a) PANI-CA-1 (b) PANI-CA-3 (c) sensor as normalized current (I/I0) to the concentration ranging from 1 to 50 ppm flowed through the measuring chamber (1000 sccm, V = +0.1 V) at 80% RH, Table S3. Repeatability data of PANI (a) PANI-CA-1 (b) PANI-CA-3 (c) sensor as normalized current (I/I0) to the same concentration of H2S (20 ppm) flowed through the measuring chamber (1000 sccm, V = +0.1 V).
