# Supplementary material for: H2S-Sensing Studies Using Interdigitated Electrode with Spin-Coated Carbon Aerogel-Polyaniline Composites
Source: Polymers (Basel). 2021 Apr 30;13(9):1457. doi: 10.3390/polym13091457 (PMC8124192; doi:10.3390/polym13091457)
Supplement: Supplementary file 1 [file polymers-13-01457-s001.zip › polymers-1168950-supplementary.pdf]

Supporting Information:

Synthesis of Polyaniline (PANI)

PANI was typically synthesized by oxidative chemical polymerization using equimolar quantity of aniline and ammonium persulphate (APS) in 1.0 *M* HCl aqueous solution. The reaction was performed in an ice bath with drop-wise addition of APS into aniline monomer solution. And then, it was stirred for 4 hours followed by vacuum filtration. The PANI-emeraldine salt thus obtained washed with 1.0 *M* HCl and distilled water. Subsequently, PANI was de-doped with 1.0 *M* ammonium hydroxide solution for further 12 hours under magnetic stirring to produce emeraldine base (EB) form of PANI. Afterwards, the EB form of PANI was filtered, washed with DI water and dried under vacuum for 24 hours. The typical yield of obtained EB form of PANI was calculated at ~ 35%.

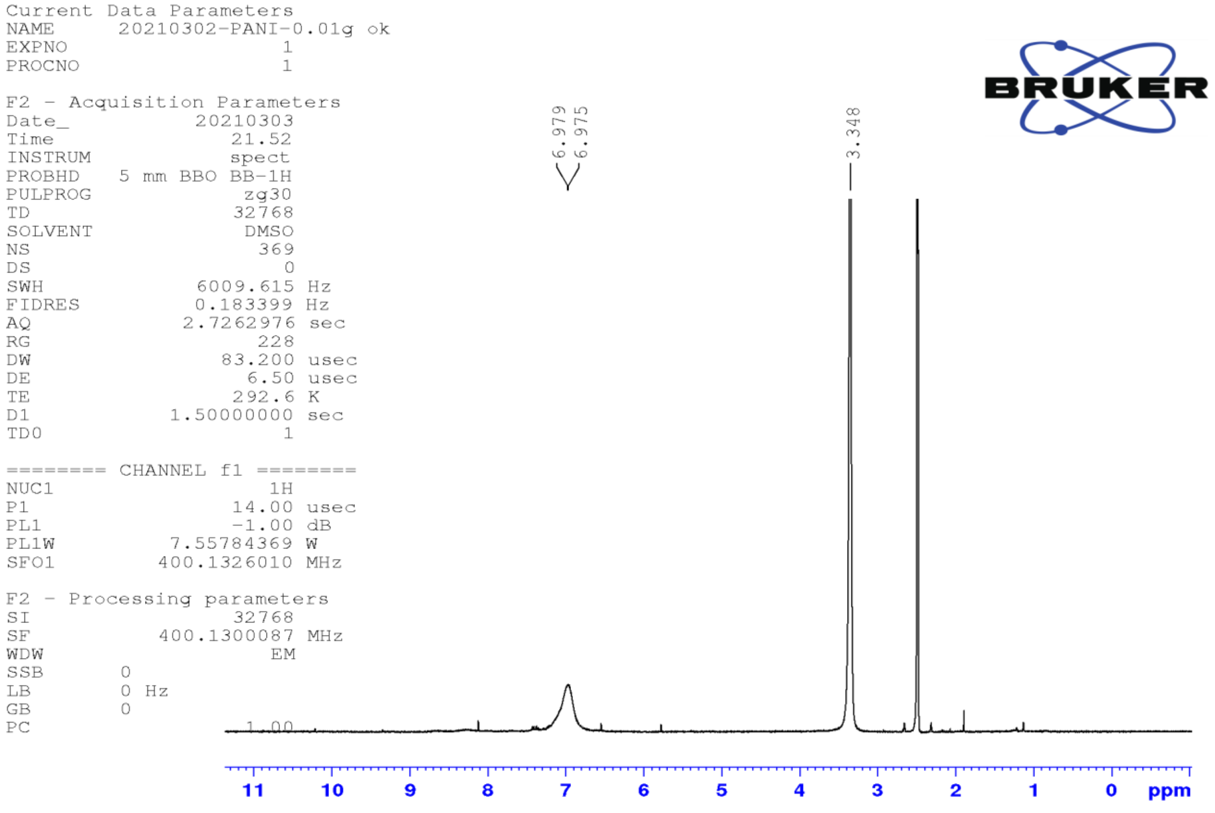

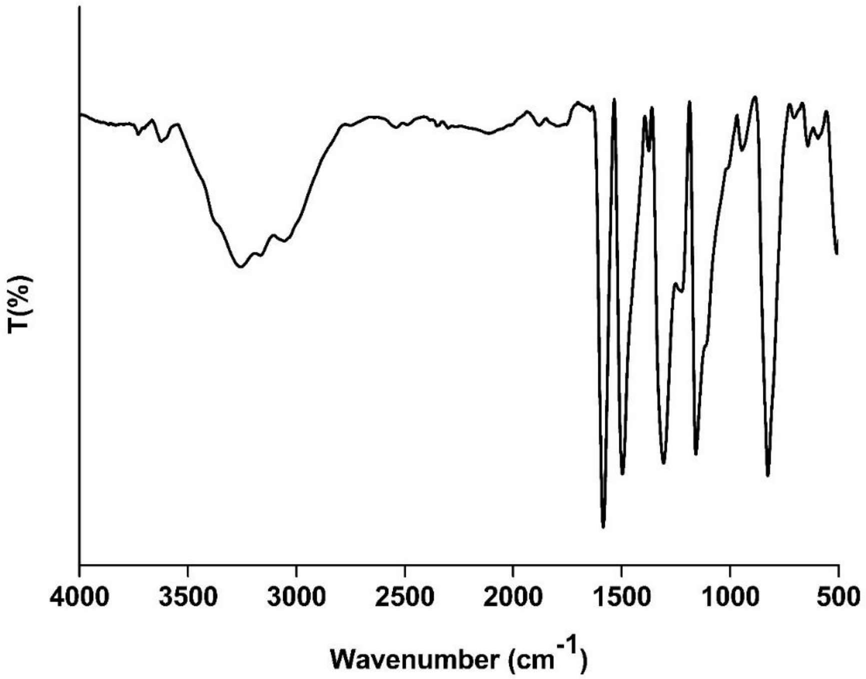

Fig. S1 The representative spectrum of (a) ^{1}H-NMR and (b) FTIR of PANI

Table S1. Quick response(s) and recovery time data of PANI (a) PANI-CA-1 (b) PANI-CA-3 (c) sensor as normalized current (I/I_0) to the concentration ranging from 1 to 50 ppm flowed through the measuring chamber (1000 sccm, V = +0.1 V) at 60% RH.

Response time (s):

Material Name	***1 ppm***	***5 ppm***	***10 ppm***	***20 ppm***	***30 ppm***	***40 ppm***	***50 ppm***
**PANI**	108	50	30	15	9	5	1
**PANI-CA-1**	1	1	1	1	1	1	1
**PANI-CA-3**	1	1	1	1	1	1	1

Recovery time (min):

**Material Name**	**1 ppm**	**5 ppm**	**10 ppm**	**20 ppm**	**30 ppm**	**40 ppm**	**50 ppm**
**PANI**	5.25	11	12.5	13.25	14.75	15.25	16.25
**PANI-CA-1**	2.25	7.75	10.25	12.25	13.75	15.25	17.75
**PANI-CA-3**	8.5	10	13	13.75	15	15.25	16.75

Table S2. Quick response(s) and recovery time data of PANI (a) PANI-CA-1 (b) PANI-CA-3 (c) sensor as normalized current (I/I_0) to the concentration ranging from 1 to 50 ppm flowed through the measuring chamber (1000 sccm, V = +0.1 V) at 80% RH

Response time (s):

Material Name	***1 ppm***	***5 ppm***	***10 ppm***	***20 ppm***	***30 ppm***	***40 ppm***	***50 ppm***
**PANI**	96	32	29	25	25	13	9
**PANI-CA-1**	72	28	26	23	20	10	5
**PANI-CA-3**	73	28	27	23	20	9	2

Recovery time (min):

**Material Name**	**1 ppm**	**5 ppm**	**10 ppm**	**20 ppm**	**30 ppm**	**40 ppm**	**50 ppm**
**PANI**	5.25	18	30	31.5	34	35	38
**PANI-CA-1**	16	21	25	28	30	32	35
**PANI-CA-3**	18	23	27.5	29	33	34	36

Table S3. Repeatability data of PANI (a) PANI-CA-1 (b) PANI-CA-3 (c) sensor as normalized current (I/I_0) to the same concentration of H_2S (20 ppm) flowed through the measuring chamber (1000 sccm, V = +0.1 V)

Sensor	***trial 1***	***trial 2***	***trial 3***	***Average***	***SD***
PANI	**6.68551**	**6.70123**	**6.69212**	**6.692953**	**0.007893**
PANI-CA-1	**12.70123**	**12.80968**	**12.90968**	**12.80686**	**0.104254**
PANI-CA-3	**13.87278**	**13.8901**	**13.90102**	**13.88797**	**0.01424**
